# Augmented reality for advanced prosthetic training in non-amputees

**DOI:** 10.1371/journal.pone.0338607

**Published:** 2026-02-27

**Authors:** Lauren Deus, Leah Wohlbach, Susan E. D’Andrea

**Affiliations:** 1 Department of Kinesiology, College of Health Sciences, University of Rhode Island, Kingston, Rhode Island, United States of America; 2 Department of Computer, Electrical and Biomedical Engineering, College of Engineering University of Rhode Island, Kingston, Rhode Island, United States of America; Roma Tre University: Universita degli Studi Roma Tre, ITALY

## Abstract

Prosthetic abandonment is highly prevalent among upper extremity amputees (UEA), partly due to lack of engaging and motivating training to use their devices. Augmented reality (AR) systems can be used for rehabilitation, and are advantageous because they offer stimulating, goal-oriented experiences for participants to immerse themselves in while performing repetitive tasks to improve function. Thus, in this study, the effects of a novel AR prosthetic training game, and the transfer of skills to a functional rehabilitation performance assessment involving tasks of daily living were investigated. Thirty-two able-bodied participants, sixteen allocated to receive AR training and sixteen received no training, donned a bypass body-powered prosthetic device to engage in a functional task assessment, evaluated at two time points. The AR group used the bypass prosthesis to engage in the AR training game, *ARm-Strong,* on each of three training sessions. AR group participants completed a questionnaire at the end of the intervention to evaluate their feelings on the AR game. On average, individuals in the AR group were significantly more efficient at using the bypass prosthesis to complete functional tasks compared to the control group, however, the rate of improvement was similar between groups. Individuals who engaged in the AR training felt positive feelings of engagement, engrossment, and immersion towards the application although the impression of immersion was significantly less than that of engagement and engrossment. The results of this study support previous findings that AR training is an engaging and motivating experience, and motor learning can be achieved through this type of training. Future implications of the results may benefit prosthesis users by enhancing user experience during prosthetic training and ultimately lead to better rehabilitation and overall adherence to the use of the prosthetic device.

## Introduction

In the United States, approximately 185,000 limb amputations are performed each year [1]. The prevalence of limb loss was approximately 2 million individuals in the year 2014 and is expected to increase one and half times that by the year 2050 [2]. Trauma-related events remain the most common cause of upper extremity amputations (UEA), primarily effecting adult males who are otherwise healthy and often return to work following recovery [3]. For individuals with UEAs, upper extremity range of motion and residual motor function is dependent on the level of limb loss. Distal amputations occurring below the elbow have less of an impact on neuromuscular performance than a shoulder or transhumeral amputation [4]. Amputations at the transradial level occur most frequently [5], which preserves elbow function despite the loss of wrist joint movements. In order for individuals with UEA to maintain high quality of life and perform tasks associated with daily living and occupational responsibilities, prosthetic devices are designed to replace the role of the missing limb.

Technology has advanced the quality of artificial limbs so that they more accurately appear and function as real limbs. However, many designs require a great deal of energy and force to be effective, which often leads to fatigue and worse, prosthesis abandonment [6]. Among the individuals with UEA who reject their body-powered prosthesis, more than 50% say they are dissatisfied due to one of three reasons: the device is uncomfortable, it has limited range of motion and capabilities, or they were not properly trained to use the device and therefore lack motivation and knowledge to be able to use their device [7,8]. While improvements in comfort and function of prostheses are necessary and desired by users, these concerns are well-documented in the literature. Prosthetic training, however, is a more ill-defined concept and unresolved issue.

It is essential for prosthesis users to receive prosthetic training, as it provides the foundation for teaching individuals how to control their device so that they may integrate using their prosthesis throughout their daily living [9]. A survey on prosthesis satisfaction of Veterans found that 23.9% of individuals with UEA never received instruction on how to use their body-powered device [10]. Consequently, those individuals who did not receive training were dissatisfied with their device. Thus, optimizing prosthetic training will benefit the population of upper extremity prosthesis users, granting them the opportunity to achieve a higher level of autonomy and contentment throughout their lives.

The principles of physical and occupational therapies for rehabilitation involve performing high repetition, task-specific exercises to increase neuroplasticity in the brain-regions related to motor function. The conventional prosthetic training methodology has resulted in successful outcomes for prosthetic users, mainly an improvement in ability to perform tasks [11–14]. However, traditional prosthetic training is monotonous and often leads individuals to develop an internal focus of attention, which can deter user’s accuracy and progress [15]. The theory of attentional focus postulates that skill training may be more effective when users have an external focus of attention (i.e., on accomplishing the task) rather than on internal thoughts (i.e., how to move to accomplish a task) [16].

Game-based training is advantageous for prosthetic training in that it directs the user’s attention externally through goal-oriented tasks resulting in increased retention and efficiency of motor learning [17,18]. Game-based approaches to rehabilitation seek to enhance the psychological commitment that is required when working to restore a person’s abilities [11–13,17,19,20]. When psychological components of therapy [such as engagement, engrossment, and immersion] are fulfilled, adherence to therapy is more likely to increase and lead to greater gains in physical function [21]. Recently, researchers have investigated the effects of serious games on prosthesis acceptance. Game-based learning combines the repetitive tasks performed in therapy sessions with a stimulating environment, which has shown improvements in adherence while still achieving higher motor function capabilities [20,22]. Particularly, advanced technologies in the form of virtual and augmented reality are being used in the healthcare setting.

Augmented reality (AR), unlike virtual reality, allows for an overlapping of virtual and real-world environments. Traditional rehabilitation supplemented with AR game-based training stimulates goal-directed behaviors by providing real-time feedback [23], which increases motivation and enjoyment towards an experience [24,25]. Augmented reality is also advantageous in that it offers virtual hand tracking capabilities, which increases user engagement and immersion within the AR environment [15,24–32]. Current games for prosthetic training allow users to adopt the controls of a virtual hand to engage with virtual adaptations of common rehabilitative measures, such as the Box and Block Test of manual dexterity and the Clothespin Relocation Test [15,24,26,27,33,34]. While these endeavors have contributed to a better understanding of AR and its potential for prosthetic training, no studies have allowed the participant to use their own prosthesis to interact with gaming elements; previous studies have only allowed participants control of virtual prostheses which appear within the game environment to manipulate gaming elements. Thus, it is unknown if users may experience greater embodiment and functional benefits from using a real prosthesis to interact with virtual elements in an AR environment.

Previous investigations of prosthetic training often recruit participants from able-bodied populations to simulate prosthetic function. While simulated studies do not directly translate to the experiences of prosthesis users, they are a reliable alternative [32]. In prosthetic training research, bypass prostheses are devices meant for able-bodied participants to simulate wearing a prosthetic. These devices are advantageous because they expand the opportunity to conduct statistically powered studies as it is often difficult to recruit amputees for participation. Additionally, bypass devices support the internal validity of research studies because the same device can be used by all participants and all able-bodied users have no prior experience using the device compared to actual amputees who often do not share the same level or type of experiences.

Therefore, bypass prostheses can be used to conduct investigations aimed at improving prosthetic training, which impacts the satisfaction of prosthesis users. Thus, the purpose of this investigation is to assess the effectiveness of an AR prosthetic training game, *ARm-Strong,* in able-bodied individuals using a bypass prosthesis. User engagement and enjoyment upon interacting with the AR game will also be evaluated. This study seeks to contribute to the literature on the effectiveness of AR for rehabilitation, specifically its physical and cognitive impact on upper limb prosthesis training.

## Materials and methods

### Participants

**Fig 1:**
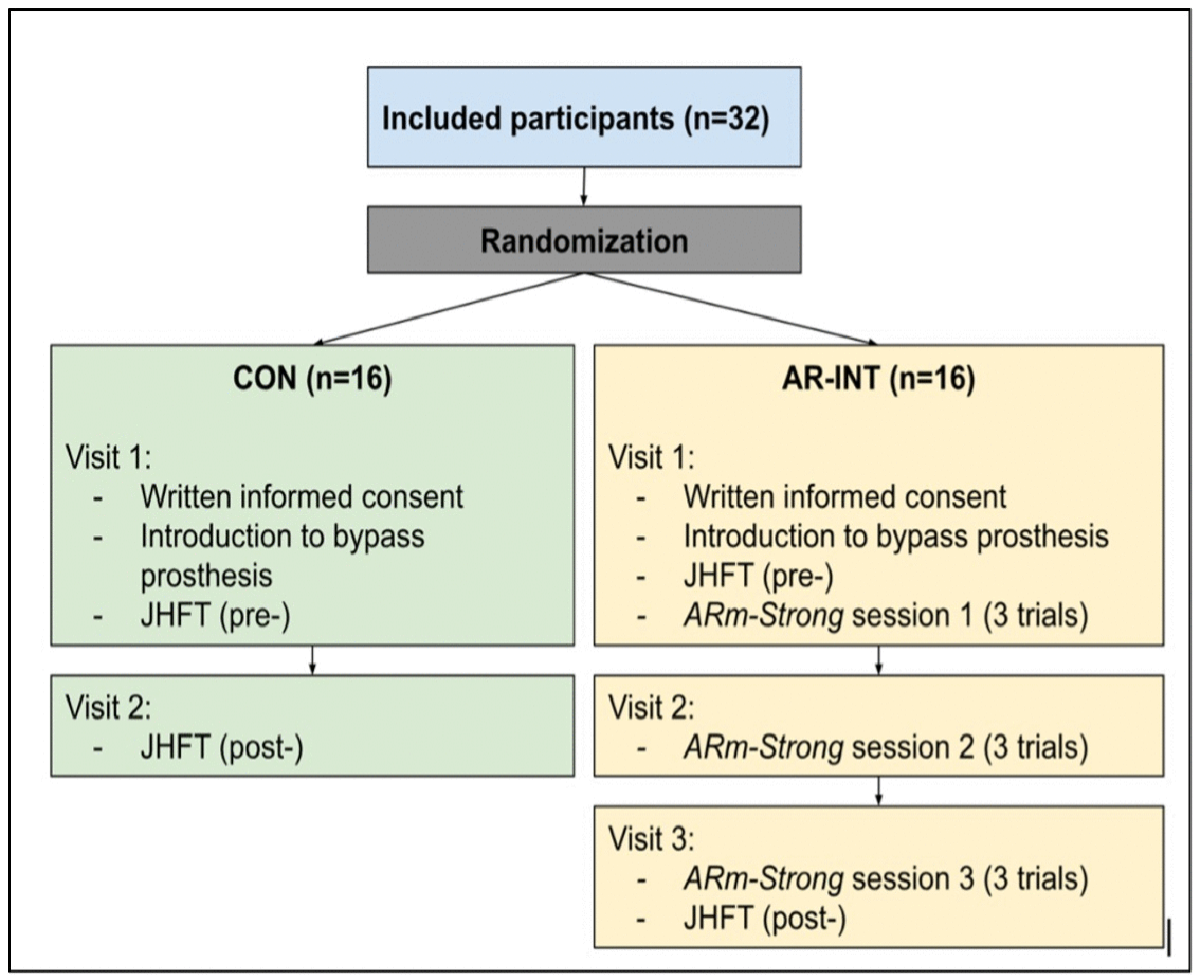
Experimental study design. ARm-Strong refers to the AR intervention.

This study was approved by the University of Rhode Island Institutional Review Board [IRB#: 1991645−2, Local Reference#: IRB2223−06]. Thirty-two able-bodied participants were recruited for this study between 11/01/2023 and 19/06/2023. A power analysis was completed and it was determined that 16 individuals per group was sufficient to reach statistical power. To be included in this investigation, participants were between the ages of 18 and 65 years, and had no prior experience using a bypass prosthesis. A diagnosis of a severe communicative or cognitive disorder, and/or susceptibility to photosensitive seizures excluded individuals from participating in this study. Participant characteristics are listed in Table 1. All participants provided written informed consent prior to participating in the study. Participants were randomly allocated to either the AR intervention group (AR-INT; n = 16) or the control group (CON; n = 16). Participants in the CON group received no training between pre- and post-measurements of hand function and were instructed not to use a prosthesis in between visits. The AR-INT group participated in the game-based training during all visits and had hand function evaluated before and after training (Fig 1). No participants had previous experience with AR.

**Table 1:** Participant Characteristics.

	CON (n = 16)	AR-INT (n = 16)
**Age; yrs** (Mean ± 1SD)	26.19 (11.71)	29.81 (10.69}
**Sex**		
Male	2	8
Female	14	8
**Handedness**		
Left	3	1
Right	13	15

### Experimental setup

**Fig 2:**
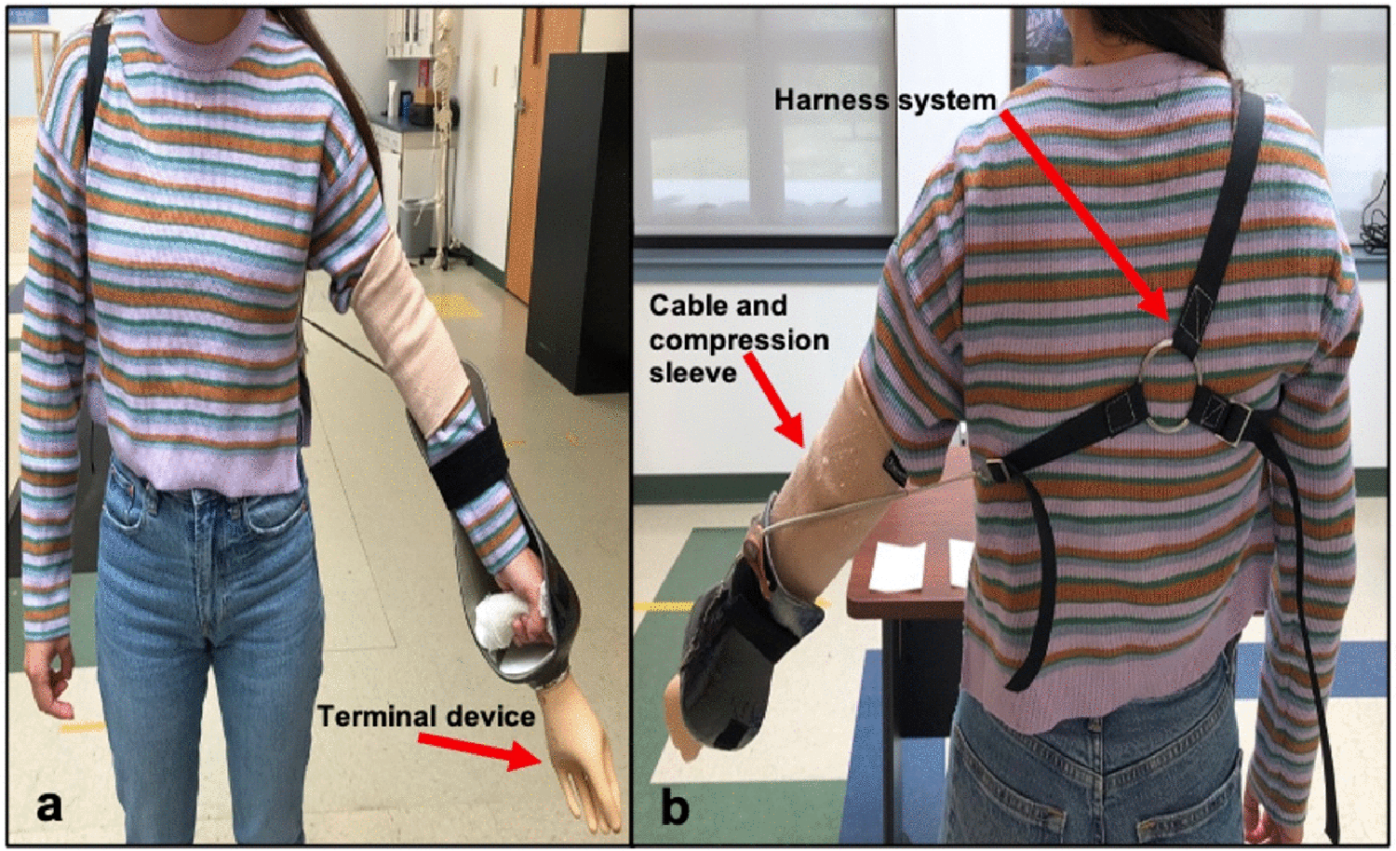
Orientation of the bypass prosthesis on a participant. **[a]** Anterior view; **[b]** Posterior view.

A body-powered bypass prosthesis, featuring a voluntary opening Otto Bock System Hand [Otto Bock; Duderstadt, Germany] terminal device, carbon-fiber socket, Bowden-cable, manual wrist rotation and harness were constructed for the investigation. Participants wore a Kunto elbow compression sleeve on their arm to prevent abrasion from the Bowden-cable. The bypass device was then attached to their left arm and the straps adjusted for each participant. To simulate prosthetic function of an amputee, able-bodied participants grab hold of a stationary grip embedded inside the socket. Motor function of the limb in use is then operated through the terminal device, which in this study was oriented distal of the participant’s hand. To operate the prosthesis, users manipulate their body movements to create tension in the cable-harness system, which causes the terminal device end to open and close (Fig 2).

### Experimental protocol

#### Control group.

Control group (CON) participants were tested during two visits, which were approximately 8–12 days apart. On the first visit, participants were fitted with the bypass prosthesis on their left arm and were familiarized with how to control the device by extending their left arm and internally rotating their shoulders to open and close the device. All participants wore the bypass prosthetic on their left arm. The left side was chosen to more realistically reflect the level of difficulty of amputees when they first learn to use a prosthetic device as the majority of the population (90% of individuals) are right-dominant [35].

**Fig 3:**
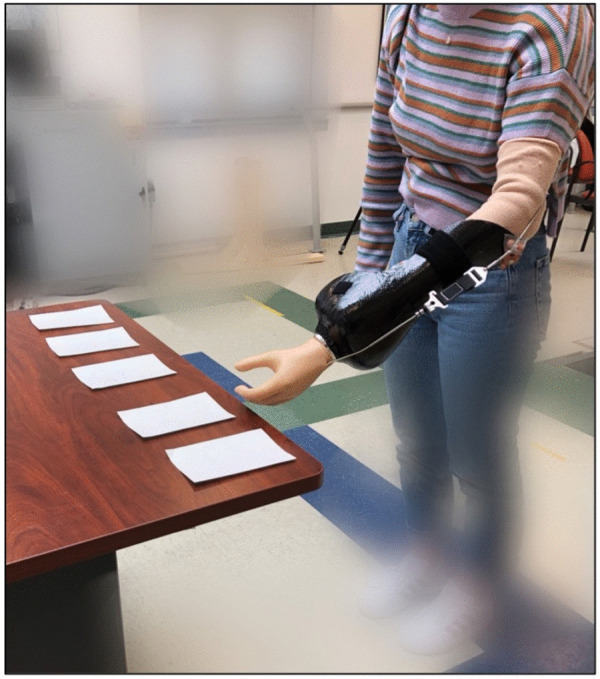
Performance of the card-turning task of the JHFT using a bypass prosthesis. A participant is wearing the bypass prosthesis oriented on their left arm. They are instructed to complete a seven-task functional assessment using the prosthesis only. The card-turning task asks that individuals turn five, 5x7 index cards over on a table in order from right to left. The time to successfully complete the task is recorded.

Baseline functional performance was assessed using the Jebsen Hand Function Test (JHFT) in the bypass device. The JHFT is a standardized, objective assessment of motor function using simulated activities of daily living [36,37]. Tasks include writing, turning over 3-by-5 inch cards (Fig 3), picking up small common objects, simulated feeding, stacking checkers, and picking up large heavy and light objects. On the second visit, participants completed a post-assessment of the JHFT using the bypass prosthesis.

#### AR-INT group.

Participants in the AR-INT group were tested during three visits; each visit was approximately 4–6 days apart. On the first visit, participants were fitted and familiarized with the bypass prosthesis and completed the baseline JHFT. Participants then donned the HoloLens and interacted with the *ARm-Strong* AR prosthetic training game to familiarize themselves with the goggles, the set up and the type of type of hand gestures needed to perform the tasks in AR. The purpose of the AR training is to encourage participants to improve motor function using a prosthesis through a goal-oriented task, which means they are not focused on their movements but rather the objective of the game. The ARm-Strong game is custom software designed in the Unity 3D gaming platform. The game is used in conjunction with the Microsoft HoloLens, an AR headset which consists of a self-contained, holographic computer that enables the user to engage with digital content with hand gestures and interact with holograms in their real-life environment. By leveraging the complex hand tracking capabilities of the HoloLens2 and the object modeling capabilities of the Unity 3D platform, the terminal devices of the upper extremity prosthetics can be used in the AR environment to provide engaging rehabilitation games for UEA prosthesis users.

**Fig 4:**
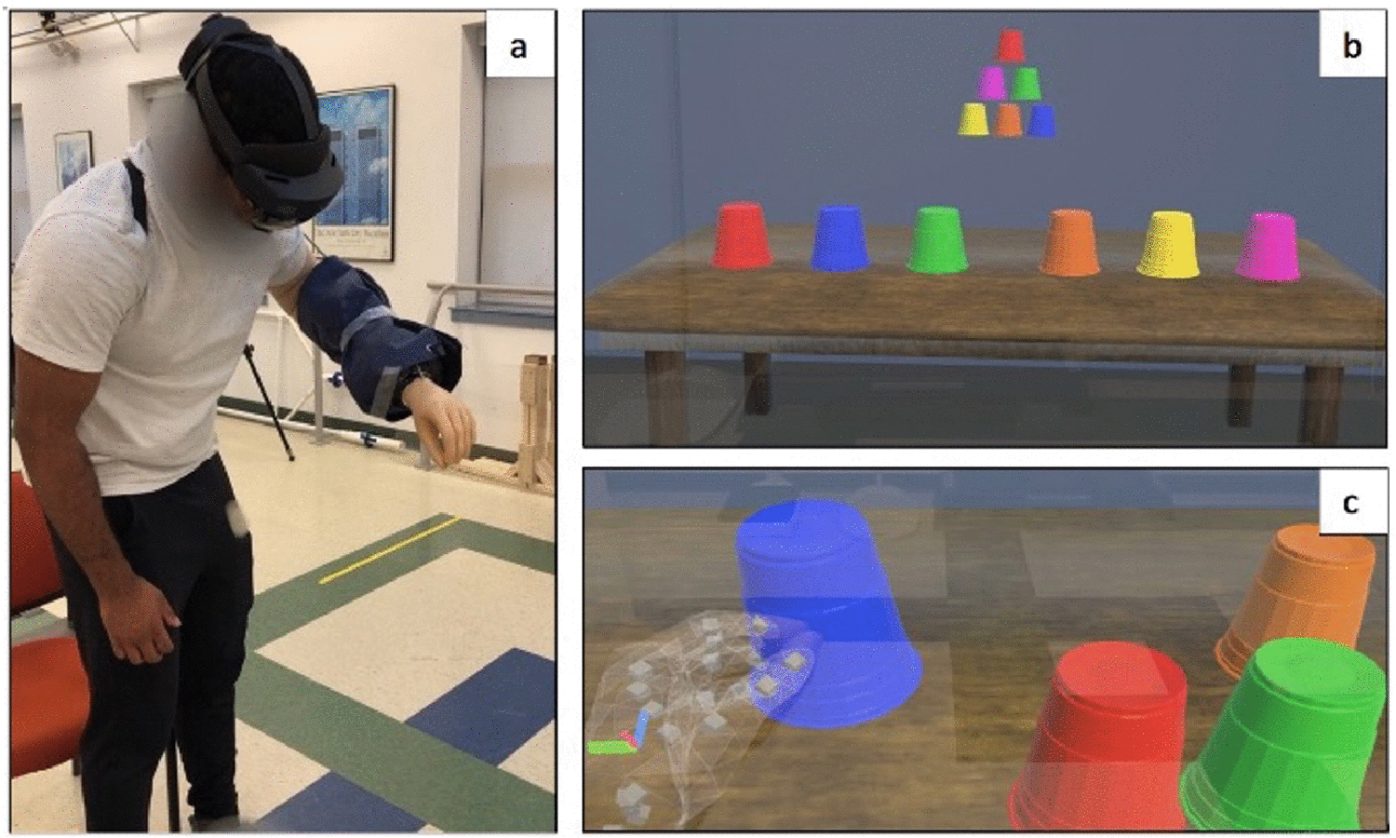
ARm-Strong prosthetic training game. **[a]** Participant wearing the bypass prosthesis and Microsoft HoloLens2 headset to play the AR training game, ARm-Strong; **[b]** ARm-Strong game setup when launching the app. Participants stack the cups on the table to match the configuration depicted in the pyramid scheme; **[c]** A Participant grasping a virtual blue cup using the bypass device.

The participants were seated and donned the HoloLens2 headset and engaged in the ARm-Strong AR game, while wearing the bypass prosthesis (Fig 4). During AR game use, users observed a holographic scene displayed in front of them consisting of six virtual plastic cups, each a different color, displayed on a virtual table. Pictured above the table and cups were six smaller cups [one of each color cup on the table], arranged in a pyramid by random color orientation, which served as the reference image. The HoloLens hand-tracking capabilities allowed participants to select and position the holographic cups using direct touch with the prosthesis, as if they were picking up a real object.

While playing the *ARm-Strong* game, participants were instructed to arrange the cups on the table to match the reference image as quickly as possible using the bypass prosthesis. AR training was done at each visit for the AR-INT group. Participants performed three trials of the game, at each of the three visits. The time to complete the cup stacking was recorded for each trial. All AR training sessions were monitored by a member of the research staff.

On the third visit after the AR training protocol, the JHFT was readministered to determine if participants had improved functional performance. Participants in the intervention group were given the validated Augmented Reality Immersion (ARI) questionnaire to assess their level of interest and motivation in the AR training [38]. The questionnaire consisted of 21 questions on the topics of engagement, engrossment, and immersion. Engagement is recognized as the first level of total immersion, in which participants have general feelings of interest towards the AR game and acknowledge its usability. The next level, engrossment, refers to a deeper emotional investment in the AR game, where one might focus their attention, senses, and thoughts more on the game than their surroundings. The highest level of total immersion that one can experience allows users to feel as if they truly exist within the AR environment, and they are un-interrupted by real world phenomena while interacting with the game [39].

### Data analysis

For the JHFT, time to complete each task was recorded in seconds. A cumulative time was calculated by adding all task times. Change in JHFT performance was calculated by finding the difference of the pre- to post-assessment cumulative times.

For the *ARm-Strong* intervention, time in seconds was recorded for each of the three trials and totaled to generate a cumulative game performance time on each visit. Change in *ARm-Strong* performance was calculated by finding the difference in total time from visit one to visit three.

Instructions for completing the ARI questionnaire were provided to all AR-INT participants. They to evaluate whether they agree or not with each statement regarding the AR training game. Scores were based on a 7-point scale ranging from “1” – totally disagree to “7” – totally agree. Average rating of the 21-item questionnaire was calculated for each participant, as well as the average rating for each sub-category – engagement, engrossment, and immersion.

### Statistical analysis

G*Power 3.1 was used to perform a power analysis to determine the necessary sample size based on a similar study which assessed functional performance as time to complete specific tasks using a bypass prosthesis [40]. The following input parameters were used: two tails, d = 1.051177, alpha = 0.05, power = 0.8. Statistical analyses were conducted using IBM SPSS Statistics version 28.0.1.1. Participant characteristics were expressed as mean ± standard deviation. A Shapiro-Wilk test was used to test for normality of the data.

To investigate the primary aim, the differences of the outcome measure, the JHFT, between two groups at multiple timepoints were compared. A two-way mixed ANOVA was calculated to assess the Group x Time interaction for the JHFT. The “group” variable was used to differentiate participants in CON and AR-INT. The “time” variable was split into pre- and post-JHFT scores and determined as the total time in seconds to complete the assessment. Using the data from participants in the AR-INT group, a Spearman’s correlation was conducted to assess the strength of a relationship between individual’s improvements during AR training (time to complete the game) with their improvements in motor function as measured by the JHFT. Participants performed three trials of the ARm-Strong game. The cumulative time to complete all three trials was determined and the difference in time between the first and last visit was calculated to assess the change in game performance. The difference in the JHFT score between the pre- and post-intervention was determined to establish if the Arm-Strong game resulted in changes in function.

To investigate the secondary aim, a second Spearman’s correlation was computed to assess the relationship between participant’s performance during AR training and their perceptions of the AR training game*.* Each participant’s change in *ARm-Strong* game performance (the difference in total time to complete the game from first to last visit) was correlated to their average rating for all questions on the ARI questionnaire. Each participant’s average rating for each topic within the questionnaire – engagement, engrossment, and immersion – was explored using two-tailed paired t-tests, to assess the difference in values between each sub-category.

## Results

Thirty-one participants (CON, n = 16; AR-INT, n = 15) were included in the analyses for this study, with the exclusion of one participant due to inability to complete the training protocol.

### Functional performance outcomes

**Fig 5:**
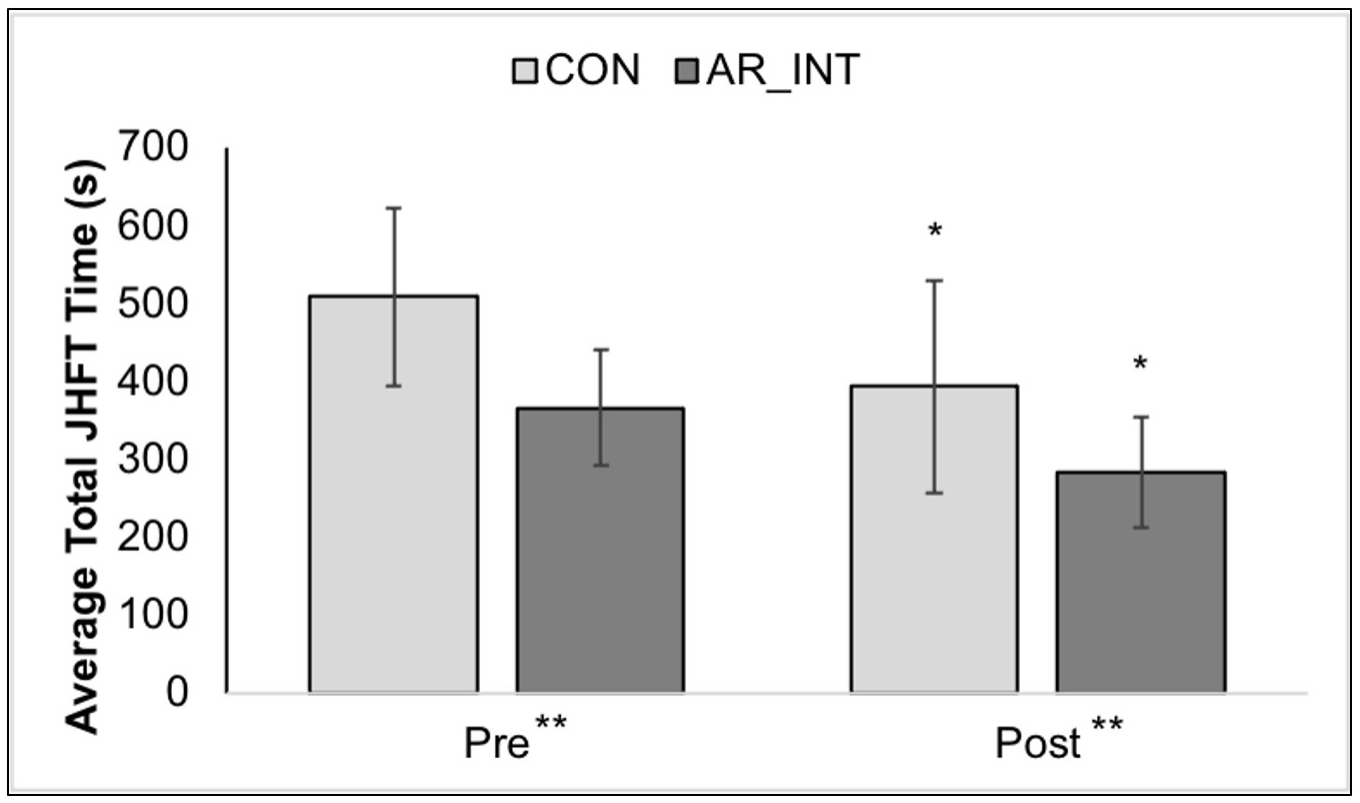
Mean ± SD of average total JHFT times assessed pre- and post-intervention in experimental (AR_INT) and control (CON) groups. Both groups significantly improved in their time to complete the JHFT from pre- to post- assessment. The AR_INT group was significantly faster at completing the JHFT compared to the CON group, at both assessment time points. Faster time [in seconds] indicates better performance. *Significant main effect of time, p < 0.05. **Significant main effect of groups, p < 0.05.

Participants in the AR-INT group completed a pre- and post-JHFT, which were on average 11.56 ± 3.31 days apart, with three sessions of AR training in between assessments. The CON group completed a pre- and post-JHFT [mean 9.31 ± 1.25 days apart], with no AR training in between assessments. The JHFT, which assesses fine and gross motor hand function using simulated activities of daily living, was used to explore potential differences in motor learning between. There were no outliers, as assessed by examination of studentized residuals for values greater than ±3 standard deviations. Data was normally distributed according to the Shapiro-Wilk test of normality (CON, p = 0.23 and 0.47; AR-INT, p = 0.27 and 0.88) at the pre- and post-intervention time points, respectively. There were improvements in motor function in both groups (Fig 5). The average decrease in time to complete the JHFT was 85.75 seconds in the intervention group and 116.09 seconds in the control group.

The two-way mixed ANOVA revealed no significant Group x Time interaction, *F*(1, 29) = 0.626, *p* = 0.435, partial n^2^ = .021, which may have been attributed to a violation of the assumption of variances, as assessed by Levene’s test, *p* < 0.05. However, there was a significant main effect of assessment time points, *F*(1, 29) = 27.70, *p* < 0.001, partial n^2^ = 0.489 and the main effect of group also showed statistical significant difference *F*(1, 29) = 17.28, *p* < 0.001, partial n^2^ = 0.373.

### Effect of augmented reality prosthetic training on physical function

**Fig 6:**
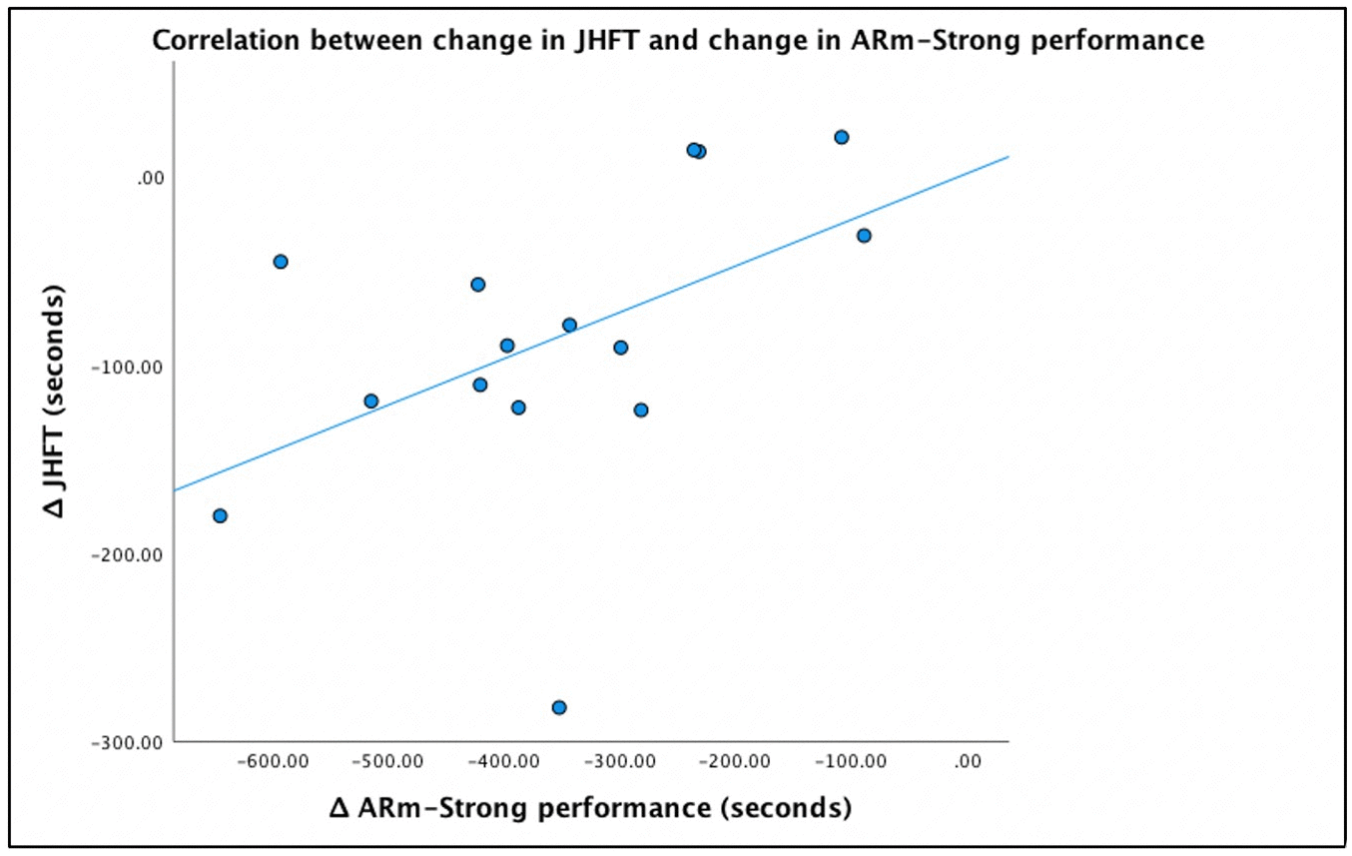
Correlation between change in JHFT and change in ARm-Strong performance. A change in ARm-Strong performance is moderately correlated with a change in JHFT performance. Participants who completed the ARm-Strong game faster also exhibited a more efficient time to complete the functional assessment, the JHFT.

To further investigate an association between AR training and functional performance outcomes in each participant in the AR-INT group, a Spearman’s rank-order correlation was used. The variables of interest were a). the difference in time from pre- to post-assessment of the JHFT, correlated with b). the change in total time to perform the *ARm-Strong* game from their first to final visit. The relationship between these variables was monotonic, as assessed by visual inspection of a scatterplot. There was a significant positive effect between variables; a greater improvement in the AR training was moderately associated with a greater improvement on the JHFT – increased quickness is indicated by a negative change in time], *r*_*s*_(13) = 0.514, *p* < 0.05 (Fig 6).

### Association between the physical effects of training and user perceptions of the augmented reality game, *ARm-Strong*

A Spearman’s rank-order correlation was run to assess the relationship between each participant’s change in total time to play the AR game [i.e., the difference in time from first to last visit] and their average rating of how involved they felt with the game represented by their average rating on the ARI questionnaire. There was a monotonic relationship, as assessed by visual inspection of a scatterplot. However, there was no statistically significant correlation between AR game performance and feelings of immersion, *r*_*s*_(13)=0.147, *p* = .602.

**Fig 7:**
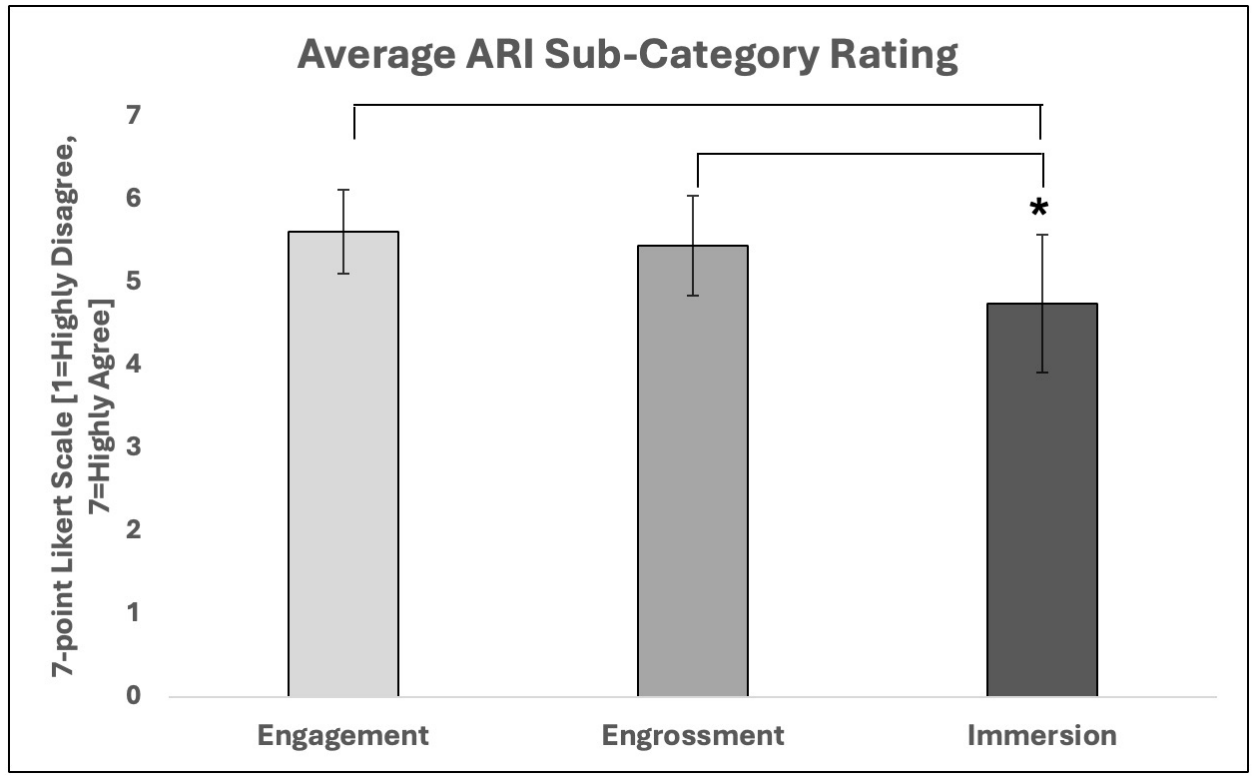
Average rating for each subcategory of the ARI questionnaire. The 21-item ARI questionnaire has questions that pertain to one of three sub-categories: engagement, engrossment and immersion. Engagement is seen as the lowest level of interest in an experience, whereas immersion is the highest level of captivation an individual can attain. Individuals who completed AR training rated their level of agreement for each question. The average ratings for each sub-category were deciphered, indicating that users felt similarly engaged and engrossed in the AR training, and did not feel as immersed. *Significant difference p < 0.05.

Each question on the ARI questionnaire pertains to one of three hierarchical sub-categories [engagement, engrossment, and immersion], which may be used to establish one’s level of total immersion. The t-tests revealed that on average, participants reported similar ratings of agreement for the sub-categories of engagement and engrossment (5.68 ± 0.74 and 5.47 ± 0.95, *p* = 0.295). When the immersion sub-category was compared to each of the first two categories, there were significant differences, indicating that individuals felt less total immersion than engagement (*p* = 0.002) and engrossment (*p* = 0.005) (Table 2 and Fig 7).

**Table 2:** Summary of differences in engagement, engrossment, and total immersion for the ARI questionnaire.

T-test	*t*	*df*	*p*
**Engagement x engrossment**	1.088	14	0.295
**Engrossment x immersion**	3.299	14	0.005*
**Engagement x immersion**	3.721	14	0.002*

Sub-categories of the ARI questionnaire.

*Significant differences between categories

## Discussion

This study explores a novel AR game for prosthetic training, which improved in-game performance as well as the transfer of skills to functional performance to complete real world tasks. Prosthetic training with the AR application demonstrated improved quickness and efficiency to complete tasks, which is a marker of motor learning. User feedback of the AR game was positive, indicating that individuals felt immersed in the experience which may indicate willingness and motivation to adhere to a training schedule which will foster better functional implementation of the prosthetic [17,18]. The results of this study support motor learning through an innovative prosthetic training system, which is important to consider for developing future patient-tailored rehabilitation protocols.

Aspects of this study showed an improved ability to complete functional tasks in both groups [those who received training and those who did not]. It is expected for motor learning to occur when individuals receive prosthetic training, which has been observed in the literature [41–44]. The present study demonstrated that the AR application, *ARm-Strong,* allows individuals to control a body-powered bypass prosthesis to interact with virtual gaming elements. Additionally, participants were able to improve their ability to operate the device upon interacting with the *ARm-Strong* training system, which we can project will enhance upper extremity prostheses users’ functional performance as well.

This study evaluated perceptions of the AR experience which is a crucial consideration regarding the practical implementation of a training protocol. The results indicate that the AR-INT group was highly engaged and engrossed during training. Previous studies have evaluated user experience after engaging with AR rehabilitation systems, and also found that AR training is an immersive experience that gives users a sense of realism [24]. The AR tasks guide users to focus on interacting with virtual objects and goals, fostering an external focus of attention that enhances motor learning based on attentional focus theory [45]. Advantages of AR training applications over conventional training have been recognized including the portability of the devices, personalized feedback to users, and increased user excitement towards the tasks [23]. The positive feedback acknowledged from studies using AR for rehabilitative training suggest that AR may lead to increased prosthesis acceptance, although this was beyond the scope of this study and should be a topic of future research. The present study has several strengths; the AR training game, *ARm-Strong,* provides users a novel experience, overlapping the virtual and real worlds in a first-person view which enhances the practicality of the training. The AR-INT group may have experienced a learning effect, which is an inherent benefit of the training and shows that motor learning was achieved. Because all participants were new to the technology, greater variability in early performance was anticipated as users adapted not only to the task demand but also the AR modality itself. Beginning the protocol with no prior AR experience, subsequent performance gains are more indicative of genuine skill acquisition, minimizing the confounding influence of AR competency and enhancing the validity of transfer and retention effects. The game experience was regarded as engaging and engrossing, which are concepts that influence adherence and improved outcomes of rehabilitation [26]. The game used in this study can be expanded upon by adding more features, such as adding additional “levels” of prosthetic training, such as flipping and rotating the cups which would involve increased range of motion with the prosthetic. The AR protocol, which only requires the AR goggles and the game software, offers flexibility for both clinical and at-home implementation, enabling upper extremity prosthetic users to receive guided, task-specific training both in and beyond the clinic.

The limitations of this study include the use of the same prosthetic device for all participants, regardless of handedness. Amputees are fitted a customized prosthesis to meet their individual needs which was not possible in the present study, although the harness was adjusted to accommodate participants. Additionally, the use of a bypass device does not directly translate to the experience of upper limb amputees, which is an aspiration for future direction. While the field of view of the HoloLens is limited, the device uses six degrees of freedom tracking which allows for the FOV to adjust to the line of vision. The Arm-Strong game is presented to the users while they are seated or standing directly in front of the AR scene making it possible for them to view the entire game. If the holographic images of the cups were “dropped” on the floor or pushed to the side during gameplay, the user was able to move their head to adjust their line of vision and focus on the fallen hologram. The three-session intervention protocol was based on the results by Huinink and colleagues who found that body-powered bypass prosthesis users were significantly quicker at operating a prosthesis after three training sessions [40]. While increased functional performance was observed in the present study after three training sessions, there was no follow-up assessment to determine the long-term effects of training which could be a consideration for future research. The AR game itself could incorporate more realistic scenes to improve the highest level of total immersion as indicated by ARI scores, although participants in this study still felt engaged and engrossed by the game.

## Conclusion

This study demonstrates that AR prosthetic training improves functional performance in individuals using a bypass prosthesis. This type of training provides users with a unique and engaging experience which can promote ongoing utilization. These findings could impact prosthetic training for amputees, by increasing motivation and thereby adherence to using their device. Developing effective mechanisms to enhance prosthesis acceptance is essential so that prosthesis users can achieve tasks in their daily life.

## Supporting information

S1 Table Jebsen Hand Function Test – Control Group. (XLSX)

S2 Table Jebsen Hand Function Test – AR-Intervention Group. (XLSX)

S3 Table Augment Reality Immersion Questionnaire Results. (XLSX)

S1 File Augmented Reality Immersion [ARI] Questionnaire. (PDF)
