# Supplementary material for: Augmented reality for advanced prosthetic training in non-amputees
Source: PLoS One. 2026 Feb 27;21(2):e0338607. doi: 10.1371/journal.pone.0338607 (PMC12948062; doi:10.1371/journal.pone.0338607)
Supplement: S1 File — (PDF) [file pone.0338607.s004.pdf]

Scoring the Augmented Reality Immersion [ARI] Questionnaire

Scoring the Levels of Immersion

Engagement: Add up the responses to all 8 “Engagement” items; responses to Q1, Q2, Q4, Q5, Q9, Q10, Q16 and Q19. Please note that responses to Q5 and Q16 should be primarily reversed (e.g. 1 becomes 7, 2 becomes 6, 3 becomes 5 etc.). Divide the sum by the number of items, namely by 8, in order to calculate the average score for “Engagement”.

Engrossment: Add up the responses to all 6 “Engrossment” items; responses to Q6, Q7, Q11, Q12, Q14, and Q17. Divide the sum by the number of items, namely by 6, in order to calculate the average score for “Engrossment”.

Total immersion: Add up the responses to all 7 “Total immersion” items; responses to Q3, Q8, Q13, Q15, Q18, Q20, and Q21. Divide the sum by the number of items, namely by 7, in order to calculate the average score for “Total immersion”.

Scoring the Components of Immersion

Interest: Add up the responses to all 4 “Interest” items; responses to Q2, Q4, Q9, and Q10. Divide the sum with the number of items, namely by 4, in order to calculate the average score for “Interest”.

Usability: Add up the responses to all 4 “Usability” items; responses to Q1, Q5, Q16, and Q19. Please note that responses to Q5 and Q16 should be primarily reversed (e.g. 1 becomes 7, 2 becomes 6, 3 becomes 5 etc.). Divide the sum with the number of items, namely by 4, in order to calculate the average score for “Usability”.

Emotional attachment: Add up the responses to all 3 “Emotional attachment” items; responses to Q11, Q14, and Q17. Divide the sum with the number of items, namely by 3, in order to calculate the average score for “Emotional attachment”.

Focus of attention: Add up the responses to all 3 “Focus of attention” items; responses to Q6, Q7, and Q12. Divide the sum with the number of items, namely by 3, in order to calculate the average score for “Focus of attention”.

Presence: Add up the responses to all 4 “Presence” items; responses to Q15, Q18, Q20, and Q21. Divide the sum with the number of items, namely by 4, in order to calculate the average score for “Presence”.

Flow: Add up the responses to all 3 “Flow” items; responses to Q3, Q8, and Q13. Divide the sum with the number of items, namely by 3, in order to calculate the average score for “Flow”.

Reference: Georgiou, Y., & Kyza, E. A. (2017). The development and validation of the ARI questionnaire: An instrument for measuring immersion in location-based augmented reality settings. *International Journal of Human Computer Studies*, 98, 24-37. doi:10.1016/j.ijhcs.2016.09.014

Name: ____

Age: ___________________ Gender:____________________________________ _

Augmented Reality Activity Evaluation

The following pages contain a number of statements for evaluating the augmented reality activity you have just participated into. Specifically, you are asked to evaluate whether you agree or not with the content of each statement. There are no "right" or "wrong" answers.

Some of the statements refer to the activity in general, while some of the statements refer specifically to the app, namely to the AR application which you employed on the mobile device (tablet).

It is important to read and evaluate carefully each statement for expressing how did you feel during the activity. Please rate how much you personally agree or disagree with these statements-how much they reflect how you feel or think personally.

Use the following scale:

"1" Totally disagree

"2" Strongly disagree

"3" Disagree

"4" No opinion

"5" Agree

"6" Strongly agree

"7" Totally agree

Make sure you have evaluated all of the 21 statements. If you change your mind about an answer, you can simply delete your choice and select another choice.

Some statements in this questionnaire are quite similar. Please, provide your honest opinion for each statement.

Reference: Georgiou, Y., & Kyza, E. A. (2017). The development and validation of the ARI questionnaire: An instrument for measuring immersion in location-based augmented reality settings. *International Journal of Human Computer Studies*, 98, 24-37. doi:10.1016/j.ijhcs.2016.09.014

Name: ____

Age: ____________________ Gender:______________________________________ _

	Totally disagree	Strongly disagree	Disagree	No opinion	Agree	Strongly Agree	Totally Agree
**1**. It was easy for me to use the AR application	1	2	3	4	5	6	7
**2.** I wanted to spend the time to complete the activity successfully	1	2	3	4	5	6	7
**3**. I didn't have any irrelevant thoughts or external distractions during the activity	1	2	3	4	5	6	7
**4**. I liked the activity because it was novel	1	2	3	4	5	6	7
**5**. I found the AR application confusing	1	2	3	4	5	6	7
**6**. I was more focused on the activity rather on any external distraction	1	2	3	4	5	6	7
**7**. If interrupted, I looked forward to returning to the activity	1	2	3	4	5	6	7
**8**. The activity became the unique and only thought occupying my mind	1	2	3	4	5	6	7
**9**. I liked the type of the activity	1	2	3	4	5	6	7
**10**. I wanted to spend time to participate in the activity	1	2	3	4	5	6	7
**11**. I was curious about how the activity would progress	1	2	3	4	5	6	7
**12**. Everyday thoughts and concerns faded out during the activity	1	2	3	4	5	6	7
**13**. I lost track of time, as if everything just stopped, and the only thing that I could think about was the activity	1	2	3	4	5	6	7
**14**. I was often excited since I felt as being part of the activity	1	2	3	4	5	6	7
**15**. The activity felt so authentic that it made me think that the virtual characters/objects existed for real	1	2	3	4	5	6	7
**16**. The AR application was unnecessarily complex	1	2	3	4	5	6	7
**17**. I often felt suspense by the activity	1	2	3	4	5	6	7
**18**. I was so involved in the activity, that in some cases I wanted to interact with the virtual characters/objects directly	1	2	3	4	5	6	7
**19**. I did not have difficulties in controlling the AR application	1	2	3	4	5	6	7

Reference: Georgiou, Y., & Kyza, E. A. (2017). The development and validation of the ARI questionnaire: An instrument for measuring immersion in location-based augmented reality settings. *International Journal of Human Computer Studies*, 98, 24-37. doi:10.1016/j.ijhcs.2016.09.014

Name: ____

Age: ____________________ Gender:____________________________________ _

	Totally disagree	Strongly disagree	Disagree	No opinion	Agree	Strongly Agree	Totally Agree
**20.** I felt that what I was experiencing was something real, instead of a fictional activity	1	2	3	4	5	6	7
**21.** I so was involved, that I felt that my actions could affect the activity	1	2	3	4	5	6	7

Reference: Georgiou, Y., & Kyza, E. A. (2017). The development and validation of the ARI questionnaire: An instrument for measuring immersion in location-based augmented reality settings. *International Journal of Human Computer Studies*, 98, 24-37. doi:10.1016/j.ijhcs.2016.09.014
